# Carotid body plastic behavior: evidence for D_2_–H_3_ receptor–receptor interactions

**DOI:** 10.3389/fphys.2024.1422270

**Published:** 2024-07-12

**Authors:** Elena Stocco, Aron Emmi, Maria Martina Sfriso, Aleksandar Tushevski, Raffaele De Caro, Veronica Macchi, Andrea Porzionato

**Affiliations:** ^1^ Section of Human Anatomy, Department of Neuroscience, University of Padova, Padua, Italy; ^2^ Department of Women’s and Children’s Health, University of Padova, Padua, Italy; ^3^ Department of Surgery, Oncology and Gastroenterology, University of Padova, Padua, Italy

**Keywords:** carotid body, dopamine D_2_ receptors, histamine H_3_ receptors, heterodimers, *in situ* PLA

## Abstract

Dopamine and histamine receptors D_2_R and H_3_R are G protein-coupled receptors (GPCRs) which can establish physical receptor–receptor interactions (RRIs), leading to homo/hetero-complexes in a dynamic equilibrium. Although D_2_R and H_3_R expression has been detected within the carotid body (CB), their possible heterodimerization has never been demonstrated. The aim of this work was to verify D_2_R and H_3_R colocalization in the CB, thus suggesting a possible interplay that, in turn, may be responsible of specific D_2_R–H_3_R antagonistic functional implications. The CBs of both Sprague–Dawley rats (n = 5) and human donors (n = 5) were dissected, and immunolocalization of D_2_R and H_3_R was performed; thereafter, *in situ* proximity ligation assay (PLA) was developed. According to experimental evidence (immunohistochemistry and double immunofluorescence), all the samples displayed positive D_2_R/H_3_R elements; hence, PLA assay followed by confocal microscopy analysis was positive for D_2_R–H_3_R RRIs. Additionally, D_2_R–H_3_R heterodimers were mainly detected in type I cells (βIII-tubulin-positive cells), but type II cells’ involvement cannot be excluded. RRIs may play a role in functional modulation of CB cells; investigating RRIs in the CB may guide toward the comprehension of its plastic changes and fine regulatory role while also unveiling their possible clinical implications.

## 1 Introduction

The carotid body (CB) is a peripheral arterial chemoreceptor at the carotid bifurcation playing a key role in sensing partial pressures of O_2_/CO_2_, pH, and metabolic changes in the arterial blood. In turn, CB type I cells (glomus cells) release growth factors (for extensive review see, Stocco et al., 2020), neurotransmitters (e.g., dopamine, acetylcholine, noradrenaline, adrenaline, serotonin, histamine, adenosine, adenosine 5’ triphosphate, glutamate, gamma-aminobutyric acid, and substance P), and neuromodulators (e.g., enkephalins, neuropeptide Y, calcitonin gene-related peptide, galanin, endothelin, bombesin, adrenomedullin, kisspeptins, and leptin) that are involved in initiating compensatory reflex adjustments to maintain homeostasis, thus preserving vital organs’ functions (Porzionato et al., 2008; Atanasova and Lazarov, 2014; Ortega-Sáenz et al., 2015; Porzionato et al., 2018; Iturriaga et al., 2021; Thakkar et al., 2023). Typically, these molecules act in an autocrine/paracrine manner on different receptors, including ionotropic and metabotropic receptors recognizable on CB type I cells, CB type II (sustentacular; glial-like) cells, and afferent nerve fibers (i.e., carotid sinus nerve afferent endings, conveying the stimulation through the glossopharyngeal nerve and petrosal ganglion (PG)) (Porzionato et al., 2018; Leonard and Nurse, 2020; Emmi et al., 2021).

Most metabotropic receptors are G protein-coupled receptors (GPCRs) that are able to establish physical receptor–receptor interactions (RRIs), leading to homo/hetero-complexes in a dynamic equilibrium; physical proximity (≤10 nm) and colocalization are the prerequisites for RRI occurrence (Agnati et al., 2005). Among the CB metabotropic receptors, the dopamine D_2_ receptor (D_2_R) and the histamine H_3_ receptor (H_3_R) are both present. Their physical/functional interplay was demonstrated by Ferrada et al. (2008) in striatal membrane preparations and mammalian transfected cells (HEK-293) by radioligand binding experiments and bioluminescence resonance energy transfer (BRET) assay, respectively. In addition, Xu and Pittenger (2023) confirmed the existence of an H_3_R–D_2_R complex, more recently in the mouse striatum, recurring to biochemical approaches and the proximity ligation assay. However, the presence of RRIs was only supposed but never assessed in the CB (Porzionato et al., 2018).

The first evidence of D_2_R in the CB was derived from biochemical and neuropharmacological studies by Mir et al. (1984) on rabbits; specifically, it was reported to be located pre- and post-synaptically to type I cells (*in situ* hybridization and reverse transcription-polymerase chain reaction) (Czyzyk-Krzeska et al., 1992; Bairam et al., 1996). Following dopamine release, as a consequence of hypoxia (Ureña et al., 1994; Montoro et al., 1996; Bairam et al., 2003), hypercapnia (Iturriaga and Alcayaga, 1998), and other stimuli, its binding to D_2_R on the type I cell decreases intracellular calcium (Benot and Lopez-Barneo, 1990); even though excitatory effects cannot be excluded (Bairam et al., 1996), dopamine mainly behaves like an inhibitory neurotransmitter in the CB, as proven by several physiological pieces of evidence (Zapata, 1975; Llados and Zapata, 1978; Bisgard et al., 1979; Goldman and Eyzaguirre, 1984). This role is mediated by D_2_R (Gauda et al., 1996). Considering that most physiological data have been obtained from CB preparations, excitatory (Iturriaga et al., 2009) and modulatory (Alcayaga et al., 1999) effects mediated by a dopamine interaction with PG neurons may also take place.

The presence of histamine receptors (H_1_R, H_2_R, and H_3_R) in the CB was first reported by Koerner et al. (2004), following reverse transcription polymerase chain reaction (RT-PCR) studies on rats. Later, several authors confirmed this evidence, further providing data on H_3_R localization, resulting in type I cells by immunohistochemistry (Del Rio et al., 2009; Lazarov et al., 2009; Thompson et al., 2010). Considering H_3_R behavior, H_3_R agonists (above all, histamine) lead to intracellular Ca^2+^ signaling inhibition, following muscarinic receptor activation in type I cells (Thompson et al., 2010), while the antagonists are responsible for increased chemosensory activity (Del Rio et al., 2009).

Currently, there is recognition of CB complexity; however, while much is known about individual neurotransmitters’ actions, there is scant information about how multiple neurotransmitters may integrate to shape the output of the CB (Thompson et al., 2010). Within this scenario, GPCRs and their capability to combine in homo-/hetero-dimers/complexes may possibly play a fundamental contributory role in determining CB function and plasticity as a consequence of development/aging and environmental stimuli (e.g., chronic intermittent/sustained hypoxia) (Porzionato et al., 2018). Existence of a RRI (A_2B_–D_2_) was first postulated in rat CB (type I cells) by Conde et al. (2008) and Conde et al. (2009); recently, through a proximity ligation assay (PLA)-based study, we also demonstrated the existence of A_2_AR–D_2_R RRI in both rat and human CB (Stocco et al., 2021). Thus, continuing the study on possible RRIs here (in accordance with the previous hypothesis (Porzionato et al., 2018)), the colocalization of D_2_R–H_3_R was analyzed to provide a deeper understanding of the behavior of this chemosensory organ, elucidating mechanisms that could also have important implications in clinical practice.

## 2 Materials and methods

### 2.1 Rat and human tissue sampling

Animal CB sampling was authorized by the ethical committee of Padua University, in agreement with the Italian Department of Health guidelines (Authorization No. 702/2016-PR of 15 July 2016); specifically, the tissues were isolated from five adult Sprague–Dawley rats soon after euthanasia.

Human CBs were collected from donated bodies enrolled within the Body Donation Program of the Section of Human Anatomy of the Department of Neuroscience of Padua University (Porzionato et al., 2012). The Section of Human Anatomy is the reference center for the Veneto region of Italy, and it has also been recognized among the reference centers at the national level for the conservation and use of donated bodies (Boscolo-Berto et al., 2023). Excision was furtherly authorized by the Italian law No. 10 of 10 February 2020, entitled *“Rules regarding the disposition of one’s body and post-mortem tissues for study, training, and scientific research purposes”* (Boscolo-Berto et al., 2020).

For this study, five adult subjects [three males, two females; mean age 63 years, standard deviation (SD) ± 2.7] without any evidence and/or reported history of chronic pulmonary and/or cardiovascular diseases were included. Eventual therapies in life with pharmacological molecules that could have altered/influenced the CB’s plasticity represented exclusion criteria for the enrollment.

After sampling (for human CB, within 30 h (h) after death, following the Italian Law directives (Porzionato et al., 2005; Porzionato et al., 2006; Porzionato et al., 2011; Stocco et al., 2021), the specimens were fixed (10% phosphate-buffered formalin for 72 h) to maintain the CBs’ morpho-structural characteristics and processed according to routine laboratory protocols for subsequent analyses.

### 2.2 Immunohistochemistry

To detect the presence of specific antigens (D_2_R and H_3_R) in CB tissue samples, immunohistochemistry was preliminarily adopted.

The paraffin-embedded rat and human carotid bifurcations, including the CB, were cut in longitudinal serial sections of 5-μm thickness. Once dewaxed, immunostaining was performed by an anti-D_2_R antibody (polyclonal rabbit antibody; ab150532, Abcam) (dilution: 1:200) and anti-H_3_R antibody (polyclonal mouse antibody; sc390140, Santa Cruz) (dilution: 1:50). Moreover, antigen retrieval occurred before both staining with low-pH (EnVision™ FLEX, Low pH, K8005) and high-pH (EnVision™ FLEX, High pH, K8012) buffers, respectively. The sections were incubated with the Dako Autostainer Plus Staining System (EnVision™ FLEX, High pH). To prove the immunostaining specificity, sections incubated without primary antibodies were also included (no immunoreactivity is expected); moreover, selectivity of the D_2_R antibody used here was previously demonstrated in the subthalamic nucleus and striatum (Emmi et al., 2022).

### 2.3 Double immunofluorescence

Double immunofluorescence was performed to localize D_2_R and H_3_R contextually.

Fluorescent immunohistochemistry was developed manually, according to previously established protocols (Emmi et al., 2022; Emmi et al., 2023). Autofluorescence was quenched with a 50 mM NH_4_Cl solution for 10 min. Sections were treated with a permeabilization and blocking solution (15% vol/vol Goat Serum, 2% wt/vol BSA, 0.25% wt/vol gelatin, and 0.2% wt/vol glycine in PBS) containing 0.5% Triton X-100 for 90 min before primary antibody incubation. Primary antibodies were diluted in the blocking solution and incubated at 4°C overnight. Specifically, the following antibodies were employed: rabbit anti-D_2_R primary antibody (dilution: 1:200) and mouse anti-H_3_R primary antibody (dilution: 1:50). Alexa-Fluor plus 488 Goat anti-Mouse secondary antibody (A32723, Thermo Fisher Scientific) and Alexa-Fluor plus 568 anti-Rabbit secondary antibody (A-11011, Thermo Fisher Scientific) were diluted 1:200 in the blocking solution, as above, and incubated for 60 min at room temperature. Hoechst 33258 was used for nuclear staining (Invitrogen, dilution: 1:10,000 in PBS) for 10 min. Slides were mounted and coverslipped with Mowiol solution (Novabiochem).

Confocal immunofluorescence z-stack images were acquired on a Zeiss800 confocal microscope equipped with ×63 oil objective. Images were acquired at a 16-bit intensity resolution over 2,048 × 2,048 pixels. Z-stack images were converted into digital maximum intensity z-projections, processed, and analyzed using ImageJ software.

### 2.4 Proximity ligation assay

PLA was adopted for detection/visualization of RRIs within the rat and human CBs. In brief, this approach is based on combinations of antibodies coupled to complementary oligonucleotides that are amplified and revealed with a fluorescent probe. When present, each protein–protein interaction appears as a red fluorescent spot.

*In situ* PLA was performed following the manufacturer’s guidelines on rat and human CB slices (5 μm in thickness); rabbit anti-D_2_R primary antibody (dilution: 1:200), mouse anti-H_3_R primary antibody (dilution: 1:50), Duolink^®^ *in situ* PLA detection kit (DUO92014, Sigma-Aldrich, St Louis, MO, USA), Duolink^®^ anti-rabbit PLUS probe (DUO92002, Sigma-Aldrich), and Duolink^®^ anti-mouse MINUS probe (DUO82040, Sigma-Aldrich) were used.

After tissue slice blocking using the Duolink^®^ blocking solution (37°C/60 min), the samples were incubated with the primary antibody (anti-D_2_R and anti-H_3_R) solutions and set up in the antibody diluent solution (room temperature (RT)/60 min); both the steps were performed within a humid chamber. Thereafter, the primary antibody solution was tapped off, and the slices were washed with the wash buffer (RT) before incubation with the anti-mouse and anti-rabbit secondary antibody-conjugated PLA probes in a pre-heated humidity chamber (37°C/60 min). After the hybridization, ligation, and amplification steps, to specifically investigate the colocalization site of the D_2_R–H_3_R heterodimer/heterocomplex with CB type I cells, the slices were rinsed in the wash buffer and incubated with anti-β III-tubulin (1:6,000) in the antibody diluent solution (Dako) (humid chamber, 4°C/overnight). Hence, after a careful wash in PBS, the sections were exposed to mouse Alexa Fluor-488 (1:100; 1 h at RT). After rinsing in PBS, the sections were mounted with the Vectashield mounting medium for fluorescence with DAPI (Vector Laboratories, Burlingame, CA, USA). A Zeiss800 confocal microscope equipped with ×63 oil objective was used for detection and acquisition of immunofluorescence and PLA signals, which appear as red dots in case of the heterodimers’ presence; for each field of view, z-stacks were acquired.

Negative controls were represented by non-conjugated primary antibodies with the Duolink^®^ Probes. The specificity of double immunolabeling was verified by replacing the primary antibodies with PBS.

PLA signals (red dots) were quantified by manual counting on z-stack images; the cell counter plugin of ImageJ was used. At least three randomly chosen fields from three slides of each animal/patient were used to determine the average density of the positive PLA elements ±SD. The percentage of red dot colocalization with βIII-tubulin-stained cells was also considered.

## 3 Results

### 3.1 D_2_R and H_3_R immunodetection in rat and human CB

Due to the problematic nature of GPCR antibodies, immunohistochemistry using primary antibodies is recommended to prove their quality before proceeding with more complex assays like PLA (Michel et al., 2009; Trifilieff et al., 2011).

**FIGURE 1:**
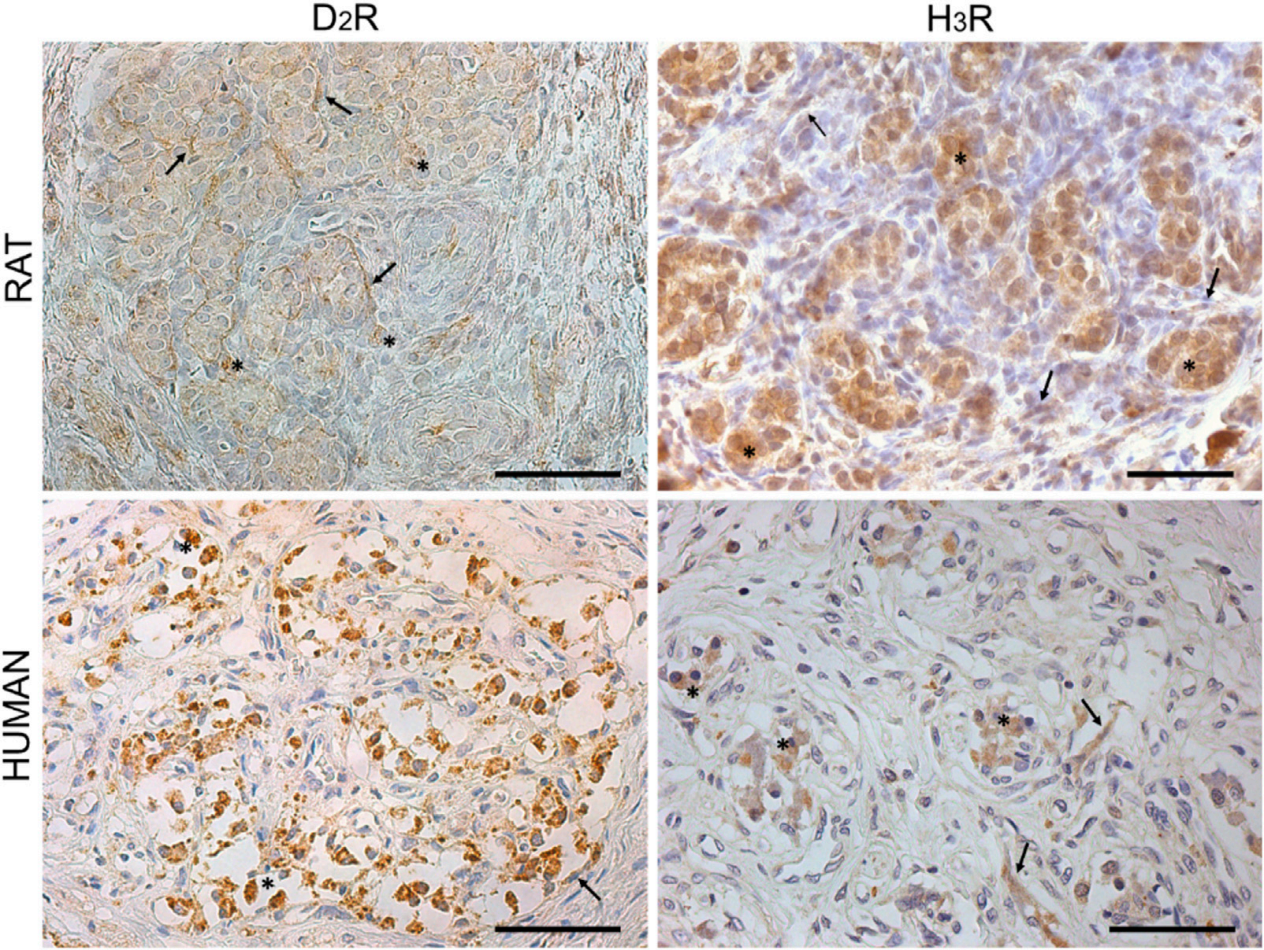
Dopamine D_2_R and histamine H_3_R detection in rat and human CB. Immunoreactive elements were mainly located in correspondence of round cells organized in clusters and, thus, resembling type I cells (black asterisk). Considering the presence of brown-stained elongated cells (black arrows), D_2_R and H_3_R were also possibly recognizable on type II cells. Scale bar: 50 μm.

Dopamine D_2_R and histamine H_3_R were preliminarily identified on rat and human CB through immunohistochemistry (Figure 1). According to the experimental evidence, brown-stained elements were mainly localized in correspondence of type I cells, which appeared as roundish elements organized in clusters. Contextually, immunoreactive elements were also observed in type II cells, displaying an elongated appearance and a peripheral position in the lobules.

Partial immunostaining of D_2_R in PG nerve terminals also cannot be excluded (Czyzyk-Krzeska et al., 1992).

### 3.2 D_2_R and H_3_R detection by double immunofluorescence

**FIGURE 2:**
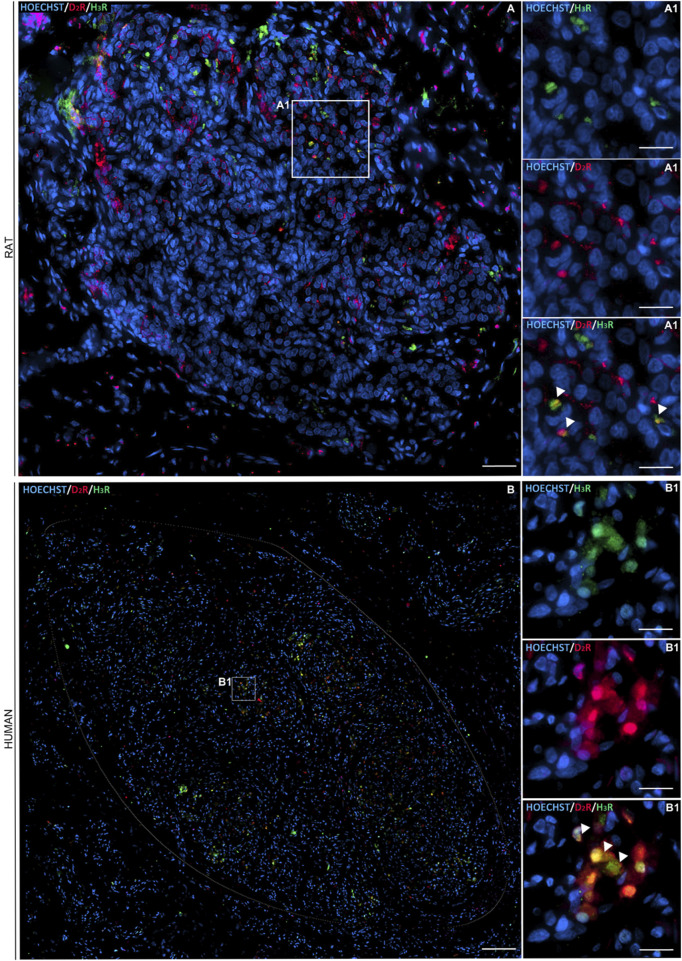
Immunofluorescence staining showing D_2_R (in red) and H_3_R (in green) distribution within a representative longitudinal section of rat and human CB (circled in white) (**A1, B1**, respectively). Cell nuclei were recognizable after Hoechst staining (in blue) (scale bar: 80 μm). The magnified images showed details in D_2_R and H_3_R localization with respect to each other and toward the cells’ nuclei (scale bar: 20 μm).

Double immunofluorescence staining for D_2_R (red channel) and H_3_R (green channel) confirmed immunohistochemistry-based evidence, highlighting the presence of both receptors within the CB. As showed by Hoechst-stained nuclei (blue channel), the receptors were localized next to them. The merged images (Hoechst/D_2_R/H_3_R) suggested a colocalization of the D_2_R and H_3_R receptors in type I cells, according to the roundish morphology of the immunopositive elements. A possible localization in type II cells also cannot be excluded (Figure 2).

Immunofluorescent elements corresponding to D_2_R may also be located at the PG nerve terminals (Czyzyk-Krzeska et al., 1992).

### 3.3 D_2_R–H_3_R heterodimer localization by PLA

**FIGURE 3:**
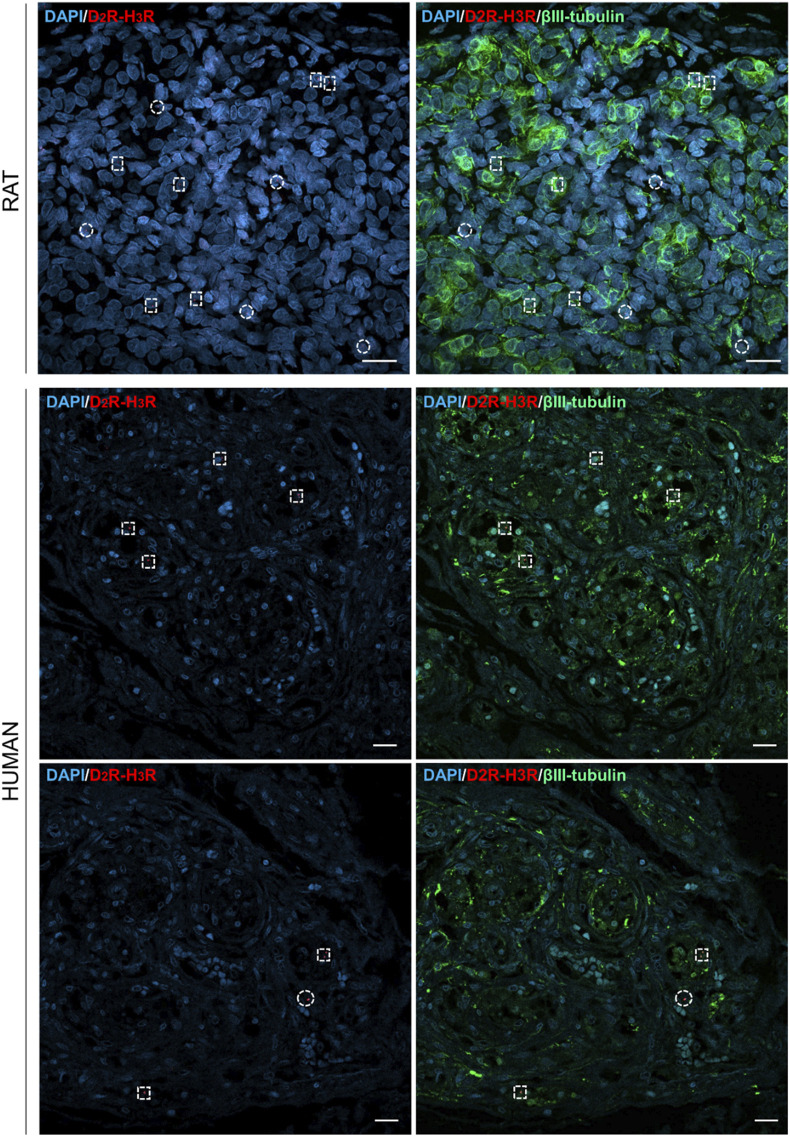
Confocal images of *in situ* proximity ligation assay showing D_2_R–H_3_R heterodimers in rat and human CBs, appearing as red dots. Anti-βIII-tubulin staining (visualized in green) allowed to detect type I cells. Cells’ nuclei were blue-stained by DAPI, and they are also recognizable in the merged images. Red dots colocalizing with type I cells were shown by dotted squares; red dots not colocalizing with type I cells were shown by dotted circles. Scale bar: 20 µm.

According to confocal microscope images, D_2_R–H_3_R complexes were verified in all the samples, thus suggesting the receptors’ closeness (distance of 0–16 nm); specifically, the red dots were identified in proximity of the DAPI-stained cells’ nuclei. The calculated mean density (±standard deviation) corresponded to 2.6 ± 0.52 × 10^−4^ heterodimers/µm^2^ and 4.90 ± 1.25 × 10^−5^ heterodimers/µm^2^ in rat and human samples, respectively. In parallel, βIII-tubulin immunostaining was also performed to recognize type I cells. According to the morphometric study, the percentages of red dots colocalizing with βIII-tubulin-positive elements in rats and humans corresponded to 54.54% and 60%, respectively (Figure 3).

## 4 Discussion

G-proteins play a crucial role in CB function both in normal physiology and pathology, possibly representing an interesting key target for novel drug discovery or repurposing. Several A GPCRs have been recognized in the CB type I/II cells (adenosine receptors (A_1_ and A_2A_), purinergic receptors (P2Y_1_, P2Y_2_, and P2Y_12_), dopamine receptors (D_1_ and D_2_), opioid receptors (μOR and δOR), histamine receptors (H_3_), serotonin receptors (5-HT_2A_), neurotensin receptors (NTS_1_), melatonin receptors (MT_1_ and MT_2_), galanin receptors (Gal_1_ and Gal_2_), endothelin receptors (ET_A_ and ET_B_), GABA receptors (GABA_B2_), muscarinic receptors (M_2_), and cannabinoid receptors (CB1) (for extensive review, see Porzionato et al., 2018)), and many of them are implicated in CB hyperactivity, thus exerting a role in conditions including obstructive sleep apnea, heart failure, and essential/spontaneous hypertension, in which the CB may be involved in promoting neurogenic hypertension and arrhythmia (Aldossary et al., 2020).

GPCRs may play a contributory role in CB high plasticity (Porzionato et al., 2018). Intramembrane receptor interactions are recognized as a functional property of some GPCR heteromers. GPCRs heterodimerization modulates the receptor function and the signaling cascade; however, heteromerization of class C GPCRs (including taste receptors and metabotropic glutamate receptors, which are obligate dimers) is well-established, and heteromerization of class A GPCRs still represents a field of intense research (Xu and Pittenger, 2023). Receptor homo- or heteromerization is responsible for intramembrane (or horizontal) interactions. It means that the pharmacology for agonists and/or antagonists of a given receptor usually changes whether (a) forming heteromers with another receptor and/or (b) when the partner receptor in the heteromer is activated. This descends from conformational changes in the receptors, which are, in turn, transmitted within the receptor–receptor interface at the plane of the membrane bilayer, as well as in the plane of the membrane (Franco et al., 2008).

In response to hypoxia, CB cells release several transmitters, and both dopamine and histamine are included (Koerner et al., 2004). This work focuses on identifying evidence supporting D_2_R–H_3_R heterodimer formation in the rat and human CB, thus laying the basis for subsequent functional studies on this chemoreceptor organ endowed with a specific plasticity. In parallel, heteromer formation is associated with different signaling and pharmacological properties, thus leading to intense research to search for novel drug targets useful for counteracting a variety of diseases and potentially with fewer side effects. Through immunodetection, D_2_R and H_3_R presence was confirmed in both rat and human CBs, corroborating previous evidence showing D_2_R immunoreactivity in mouse, rat, and human CB (Lazarov et al., 2009; Fagerlund et al., 2010; Kåhlin et al., 2010; Wakai et al., 2015) and H_3_R immunoreactivity in rat and human CB (Lazarov et al., 2006; Lazarov et al., 2009); certainly, D_2_R presence in PG neurons innervating the CB cannot be excluded (Czyzyk-Krzeska et al., 1992). In addition, for the first time, to the best of our knowledge, D_2_R and H_3_R RRIs were assessed here by PLA. As for D_2_R–H_3_R heterodimer localization, they were mainly detected in type I cells (βIII-tubulin-positive cells), but type II cells’ involvement is also possible. Moreover, for completeness, it must be mentioned that neurons and terminals of PG express β-III tubulin as well; it follows that we cannot ignore possible β-III tubulin positivity by them, even though the roundish morphology suggests that these are CB type I cells.

Ferrada et al. (2008) demonstrated the existence of an antagonistic intramembrane interaction between H_3_R and D_2_R in striatal tissue, by which, stimulation of H_3_R significantly decreases the ability of an agonist, but not an antagonist, to bind to the D_2_R. The same could also be supposed for the CB, following D_2_R–H_3_R heterodimer formation. The reciprocal influences of the two receptor monomers in the D_2_R/H_3_R complex would be particularly intriguing as dopamine and histamine (together with adenosine triphosphate (ATP) and acetylcholine (ACh) (Zhang et al., 2000; Iturriaga and Alcayaga, 2004; Leonard et al., 2018)) are among the main inhibitory and excitatory neurotransmitters in the CB. H_3_R ligands reduce the affinity of D_2_R ligands, favoring an excitatory response. In parallel, D_2_R agonists decrease the binding of H_3_R ligands, increasing the inhibitory activity of dopamine. This interpretation falls within the “push–pull mechanism” proposed to describe the role of excitatory/inhibitory transmitters involved in regulation of CB activity (Prabhakar, 2006).

Hypoxia also influences D_2_R expression (as well as D_1_R) (Bairam et al., 2003). RT-PCR analysis of short- and long-term hypoxic rats’ CBs showed a time-dependent increase in the expression of both tyrosine hydroxylase and D_2_R genes (Huey and Powell, 2000; Wakai et al., 2010). D_2_R mRNA levels decreased after 48 h of hypoxia, but a significant increase was detected after 7 days (Huey and Powell, 2000). As we previously discussed in Porzionato et al. (2018), the alteration in D_2_R expression may cause a change in dopamine signaling in the CB, contributing, for instance, to the changes in ventilatory adaptation observed with long-term hypoxia associated with chronic obstructive pulmonary disease or heart failure. Moreover, modifications in the expression of dopamine receptors after hypoxia (D_1_R and D_2_R) suggest the possibility of changes in the amount of heterodimers involving them (D_1_R–D_2_R; D_2_R–H_3_R?) stimulated by a hypoxic environment and favored by the receptors’ proximity. Other conditions also impact CB D_2_R expression; for instance, hypothyroidism induces their increase (as also verified in the paraventricular hypothalamic nucleus and striatum), in turn affecting ventilation. D_2_R stimulation and hypoxia depressed breathing in normal hamsters and stimulated breathing in hypothyroid hamsters (Schlenker and Schultz, 2012). Neonatal caffeine treatment enhances D_2_R mRNA (as well as adenosine A_2A_ and tyrosine hydroxylase) in male but not in female rats (Montandon et al., 2008); neonatal maternal separation enhances D_2_R (and tyrosine hydroxylase) mRNA expression levels in the CB of rats (not in a gender-specific manner) (Kinkead et al., 2005). Contextually, according to our knowledge, no studies have reported on H_3_R level modifications in the CB.

Knowledge of the physiological and pathological events determining the establishment of D_2_R–H_3_R heterodimers may be fundamental to provide personalized treatment of CB-mediated diseases (e.g., cardiovascular and respiratory). Furthermore, exploring the role of age on receptors’ expression and, thus, on RRI establishment may be interesting.

Future perspectives of the work will focus on other methods to better describe D_2_R–H_3_R RRIs within the CB tissue, including biophysical (e.g., bioluminescence—and fluorescence—resonance energy transfer, specialized microscopic techniques, and X-ray crystallography) and biochemical analyses. Additionally, the existence of other possible CB heterodimers may be investigated for better knowledge on the structural/functional modifications of the CB (Porzionato et al., 2018).

## Data Availability

The original contributions presented in the study are included in the article/Supplementary Material; further inquiries can be directed to the corresponding author.

## References

[B1] Agnati L. F. Fuxe K. Torvinen M. Genedani S. Franco R. Watson S. (2005). New methods to evaluate colocalization of fluorophores in immunocytochemical preparations as exemplified by a study on A2A and D2 receptors in Chinese hamster ovary cells. J. Histochem Cytochem 53, 941–953. 10.1369/jhc.4A6355.2005 16055748

[B2] Alcayaga J. Varas R. Arroyo J. Iturriaga R. Zapata P. (1999). Dopamine modulates carotid nerve responses induced by acetylcholine on the cat petrosal ganglion *in vitro* . Brain Res. 831, 97–103. 10.1016/s0006-8993(99)01402-x 10411987

[B3] Aldossary H. S. Alzahrani A. A. Nathanael D. Alhuthail E. A. Ray C. J. Batis N. (2020). G-Protein-Coupled receptor (GPCR) signaling in the carotid body: roles in hypoxia and cardiovascular and respiratory disease. Int. J. Mol. Sci. 21, 6012. 10.3390/ijms21176012 32825527 PMC7503665

[B4] Atanasova D. Y. Lazarov N. E. (2014). Expression of neurotrophic factors and their receptors in the carotid body of spontaneously hypertensive rats. Respir. Physiol. Neurobiol. 202, 6–15. 10.1016/j.resp.2014.06.016 25034384

[B5] Bairam A. Carroll J. L. Labelle Y. Khandjian E. W. (2003). Differential changes in dopamine D2-and D1-receptor mRNA levels induced by hypoxia in the arterial chemoreflex pathway organs in one-day-old and adult rabbits. Biol. Neonate. 84, 222–231. 10.1159/000072306 14504446

[B6] Bairam A. Dauphin C. Rousseau F. Khandjian E. W. (1996). Expression of dopamine D2-receptor mRNA isoforms at the peripheral chemoreflex afferent pathway in developing rabbits. Am. J. Respir. Cell Mol. Biol. 15, 374–381. 10.1165/ajrcmb.15.3.8810642 8810642

[B7] Benot A. R. Lopez-Barneo J. (1990). Feedback inhibition of Ca^2+^ currents by dopamine in glomus cells of the carotid body. Eur. J. Neurosci. 2, 809–812. 10.1111/j.1460-9568.1990.tb00473.x 12106283

[B8] Bisgard G. E. Mitchell R. A. Herbert D. A. (1979). Effects of dopamine, norepinephrine and 5-hydroxytryptamine on the carotid body of the dog. Respir. Physiol. 37, 61–80. 10.1016/0034-5687(79)90092-6 451374

[B9] Boscolo-Berto R. Porzionato A. Stecco C. Macchi V. De Caro R. (2020). Body donation in Italy: lights and shadows of law No. 10/2020. Clin. Anat. 33, 950–959. 10.1002/ca.23623 32427400

[B10] Boscolo-Berto R. Porzionato A. Stecco C. Macchi V. De Caro R. (2023). Reference centers for tissue and body donations: compulsory requirements in Italy. Clin. Anat. 36, 465–470. 10.1002/ca.23990 36514860

[B11] Conde S. V. Gonzalez C. Batuca J. R. Monteiro E. C. Obeso A. (2008). An antagonistic interaction between A2B adenosine and D2 dopamine receptors modulates the function of rat carotid body chemoreceptor cells. J. Neurochem. 107, 1369–1381. 10.1111/j.1471-4159.2008.05704.x 18823369

[B12] Conde S. V. Obeso A. Monteiro E. C. Gonzalez C. (2009). The A(2B)-D(2) receptor interaction that controls carotid body catecholamines release locates between the last two steps of hypoxic transduction cascade. Adv. Exp. Med. Biol. 648, 161–168. 10.1007/978-90-481-2259-2_18 19536477

[B13] Czyzyk-Krzeska M. F. Lawson E. E. Millhorn D. E. (1992). Expression of D2 dopamine receptor mRNA in the arterial chemoreceptor afferent pathway. J. Auton. Nerv. Syst. 41, 31–39. 10.1016/0165-1838(92)90124-y 1362730

[B14] Del Rio R. Moya E. A. Alcayaga J. Iturriaga R. (2009). Evidence for histamine as a new modulator of carotid body chemoreception. Adv. Exp. Med. Biol. 648, 177–184. 10.1007/978-90-481-2259-2_20 19536479

[B15] Emmi A. Antonini A. Sandre M. Baldo A. Contran M. Macchi V. (2022). Topography and distribution of adenosine A_2A_ and dopamine D_2_ receptors in the human Subthalamic Nucleus. Front. Neurosci. 16, 945574. 10.3389/fnins.2022.945574 36017181 PMC9396224

[B16] Emmi A. Porzionato A. Contran M. De Rose E. Macchi V. De Caro R. (2021). 3D reconstruction of the morpho-functional topography of the human vagal trigone. Front. Neuroanat. 15, 663399. 10.3389/fnana.2021.663399 33935659 PMC8085322

[B17] Emmi A. Sandre M. Russo F. P. Tombesi G. Garrì F. Campagnolo M. (2023). Duodenal alpha-synuclein pathology and enteric gliosis in advanced Parkinson's disease. Mov. Disord. 38, 885–894. 10.1002/mds.29358 36847308

[B18] Fagerlund M. J. Kahlin J. Ebberyd A. Schulte G. Mkrtchain S. Eriksson L. I. (2010). The human carotid body: expression of oxygen sensing and signaling genes of relevance for anesthesia. Anesthesiology 113, 1270–1279. 10.1097/ALN.0b013e3181fac061 20980909

[B19] Ferrada C. Ferré S. Casadó V. Cortés A. Justinova Z. Barnes C. (2008). Interactions between histamine H3 and dopamine D2 receptors and the implications for striatal function. Neuropharmacology 55, 190–197. 10.1016/j.neuropharm.2008.05.008 18547596 PMC2435196

[B20] Franco R. Casadó V. Cortés A. Mallol J. Ciruela F. Ferré S. (2008). G-protein-coupled receptor heteromers: function and ligand pharmacology. Br. J. Pharmacol. 153, S90–S98. 10.1038/sj.bjp.0707571 18037920 PMC2268068

[B21] Gauda E. B. Bamford O. Gerfen C. R. (1996). Developmental expression of tyrosine hydroxylase, D2-dopamine receptor and substance P genes in the carotid body of the rat. Neuroscience 75, 969–977. 10.1016/0306-4522(96)00312-0 8951888

[B22] Goldman W. F. Eyzaguirre C. (1984). The effect of dopamine on glomus cell membranes in the rabbit. Brain Res. 321, 337–340. 10.1016/0006-8993(84)90189-6 6093937

[B23] Huey K. A. Powell F. L. (2000). Time-dependent changes in dopamine D(2)-receptor mRNA in the arterial chemoreflex pathway with chronic hypoxia. Brain Res. Mol. Brain Res. 75, 264–270. 10.1016/s0169-328x(99)00321-6 10686347

[B24] Iturriaga R. Alcayaga J. (1998). Effects of CO2-HCO3- on catecholamine efflux from cat carotid body. J. Appl. Physiol. 84, 60–68. 10.1152/jappl.1998.84.1.60 9451618

[B25] Iturriaga R. Alcayaga J. (2004). Neurotransmission in the carotid body: transmitters and modulators between glomus cells and petrosal ganglion nerve terminals. Brain Res. Brain Res. Rev. 47, 46–53. 10.1016/j.brainresrev.2004.05.007 15572162

[B26] Iturriaga R. Alcayaga J. Chapleau M. W. Somers V. K. (2021). Carotid body chemoreceptors: physiology, pathology, and implications for health and disease. Physiol. Rev. 101, 1177–1235. 10.1152/physrev.00039.2019 33570461 PMC8526340

[B27] Iturriaga R. Alcayaga J. Gonzalez C. (2009). Neurotransmitters in carotid body function: the case of dopamine--invited article. Adv. Exp. Med. Biol. 648, 137–143. 10.1007/978-90-481-2259-2_16 19536475

[B28] Kåhlin J. Eriksson L. I. Ebberyd A. Fagerlund M. J. (2010). Presence of nicotinic, purinergic and dopaminergic receptors and the TASK-1 K+-channel in the mouse carotid body. Respir. Physiol. Neurobiol. 172, 122–128. 10.1016/j.resp.2010.05.001 20452469

[B29] Kinkead R. Joseph V. Lajeunesse Y. Bairam A. (2005). Neonatal maternal separation enhances dopamine D(2)-receptor and tyrosine hydroxylase mRNA expression levels in carotid body of rats. Can. J. Physiol. Pharmacol. 83, 76–84. 10.1139/y04-106 15759053

[B30] Koerner P. Hesslinger C. Schaefermeyer A. Prinz C. Gratzl M. (2004). Evidence for histamine as a transmitter in rat carotid body sensor cells. J. Neurochem. 91, 493–500. 10.1111/j.1471-4159.2004.02740.x 15447682

[B31] Lazarov N. Rozloznik M. Reindl S. Rey-Ares V. Dutschmann M. Gratzl M. (2006). Expression of histamine receptors and effect of histamine in the rat carotid body chemoafferent pathway. Eur. J. Neurosci. 24, 3431–3444. 10.1111/j.1460-9568.2006.05241.x 17229092

[B32] Lazarov N. E. Reindl S. Fischer F. Gratzl M. (2009). Histaminergic and dopaminergic traits in the human carotid body. Respir. Physiol. Neurobiol. 165, 131–136. 10.1016/j.resp.2008.10.016 19022410

[B33] Leonard E. M. Nurse C. A. (2020). Expanding role of dopaminergic inhibition in hypercapnic responses of cultured rat carotid body cells: involvement of type II glial cells. Int. J. Mol. Sci. 21, 5434. 10.3390/ijms21155434 32751703 PMC7432366

[B34] Leonard E. M. Salman S. Nurse C. A. (2018). Sensory processing and integration at the carotid body tripartite synapse: neurotransmitter functions and effects of chronic hypoxia. Front. Physiol. 9, 225. 10.3389/fphys.2018.00225 29615922 PMC5864924

[B35] Llados F. Zapata P. (1978). Effects of dopamine analogues and antagonists on carotid body chemosensors *in situ* . J. Physiol. 274, 487–499. 10.1113/jphysiol.1978.sp012162 625005 PMC1282505

[B36] Michel M. C. Wieland T. Tsujimoto G. (2009). How reliable are G-protein-coupled receptor antibodies? Naunyn Schmiedeb. Arch. Pharmacol. 379, 385–388. 10.1007/s00210-009-0395-y 19172248

[B37] Mir A. K. McQueen D. S. Pallot D. J. Nahorski S. R. (1984). Direct biochemical and neuropharmacological identification of dopamine D2-receptors in the rabbit carotid body. Brain Res. 291, 273–283. 10.1016/0006-8993(84)91259-9 6320958

[B38] Montandon G. Bairam A. Kinkead R. (2008). Neonatal caffeine induces sex-specific developmental plasticity of the hypoxic respiratory chemoreflex in adult rats. Am. J. Physiol. Regul. Integr. Comp. Physiol. 295, R922–R934. 10.1152/ajpregu.00059.2008 18596110

[B39] Montoro R. J. Ureña J. Fernández-Chacón R. Alvarez de Toledo G. López-Barneo J. (1996). Oxygen sensing by ion channels and chemotransduction in single glomus cells. J. Gen. Physiol. 107, 133–143. 10.1085/jgp.107.1.133 8741735 PMC2219248

[B40] Ortega-Sáenz P. Villadiego J. Pardal R. Toledo-Aral J. J. Lopez-Barneo J. (2015). Neurotrophic properties, chemosensory responses and neurogenic niche of the human carotid body. Adv. Exp. Med. Biol. 860, 139–152. 10.1007/978-3-319-18440-1_16 26303476

[B41] Porzionato A. Macchi V. Belloni A. S. Parenti A. De Caro R. (2006). Adrenomedullin immunoreactivity in the human carotid body. Peptides 27, 69–73. 10.1016/j.peptides.2005.07.017 16154664

[B42] Porzionato A. Macchi V. Guidolin D. Parenti A. Ferrara S. D. De Caro R. (2005). Histopathology of carotid body in heroin addiction. Possible chemosensitive impairment. Histopathology 46, 296–306. 10.1111/j.1365-2559.2005.02060.x 15720415

[B43] Porzionato A. Macchi V. Parenti A. De Caro R. (2008). Trophic factors in the carotid body. Int. Rev. Cell Mol. Biol. 269, 1–58. 10.1016/S1937-6448(08)01001-0 18779056

[B44] Porzionato A. Macchi V. Stecco C. Mazzi A. Rambaldo A. Sarasin G. (2012). Quality management of body donation Program at the university of Padova. Anat. Sci. Educ. 5, 264–272. 10.1002/ase.1285 22573575

[B45] Porzionato A. Rucinski M. Macchi V. Stecco C. Castagliuolo I. Malendowicz L. K. (2011). Expression of leptin and leptin receptor isoforms in the rat and human carotid body. Brain Res. 1385, 56–67. 10.1016/j.brainres.2011.02.028 21334312

[B46] Porzionato A. Stocco E. Guidolin D. Agnati L. Macchi V. De Caro R. (2018). Receptor-receptor interactions of G protein-coupled receptors in the carotid body: a working hypothesis. Front. Physiol. 9, 697. 10.3389/fphys.2018.00697 29930516 PMC6000251

[B47] Prabhakar N. R. (2006). O_2_ sensing at the mammalian carotid body: why multiple O_2_ sensors and multiple transmitters? Exp. Physiol. 91, 17–23. 10.1113/expphysiol.2005.031922 16239252

[B48] Schlenker E. H. Schultz H. D. (2012). Hypothyroidism stimulates D2 receptor-mediated breathing in response to acute hypoxia and alters D2 receptors levels in carotid bodies and brain. Respir. Physiol. Neurobiol. 180, 69–78. 10.1016/j.resp.2011.10.013 22051191 PMC3242856

[B49] Stocco E. Barbon S. Tortorella C. Macchi V. De Caro R. Porzionato A. (2020). Growth factors in the carotid body-an update. Int. J. Mol. Sci. 21, 7267. 10.3390/ijms21197267 33019660 PMC7594035

[B50] Stocco E. Sfriso M. M. Borile G. Contran M. Barbon S. Romanatoc F. (2021). Experimental evidence of a2a-D_2_ receptor-receptor interactions in the rat and human carotid body. Front. Physiol. 12, 645723. 10.3389/fphys.2021.645723 33935801 PMC8082109

[B51] Thakkar P. Pauza A. G. Murphy D. Paton J. F. R. (2023). Carotid body: an emerging target for cardiometabolic co-morbidities. Exp. Physiol. 108, 661–671. 10.1113/EP090090 36999224 PMC10988524

[B52] Thompson C. M. Troche K. Jordan H. L. Barr B. L. Wyatt C. N. (2010). Evidence for functional, inhibitory, histamine H3 receptors in rat carotid body type I cells. Neurosci. Lett. 471, 15–19. 10.1016/j.neulet.2009.12.077 20056131

[B53] Trifilieff P. Rives M. L. Urizar E. Piskorowski R. A. Vishwasrao H. D. Castrillon J. (2011). Detection of antigen interactions *ex vivo* by proximity ligation assay: endogenous dopamine D2-adenosine A2A receptor complexes in the striatum. Biotechniques 51, 111–118. 10.2144/000113719 21806555 PMC3642203

[B54] Ureña J. Fernández-Chacón R. Benot A. R. Alvarez de Toledo G. A. López-Barneo J. (1994). Hypoxia induces voltage-dependent Ca^2+^ entry and quantal dopamine secretion in carotid body glomus cells. Proc. Natl. Acad. Sci. U. S. A. 91, 10208–10211. 10.1073/pnas.91.21.10208 7937863 PMC44987

[B55] Wakai J. Kizaki K. Yamaguchi-Yamada M. Yamamoto Y. (2010). Differences in tyrosine hydroxylase expression after short-term hypoxia, hypercapnia or hypercapnic hypoxia in rat carotid body. Respir. Physiol. Neurobiol. 173, 95–100. 10.1016/j.resp.2010.07.003 20620242

[B56] Wakai J. Takayama A. Yokoyama T. Nakamuta N. Kusakabe T. Yamamoto Y. (2015). Immunohistochemical localization of dopamine D2 receptor in the rat carotid body. Acta histochem. 117, 784–789. 10.1016/j.acthis.2015.07.007 26272445

[B57] Xu J. Pittenger C. (2023). The histamine H3 receptor modulates dopamine D2 receptor-dependent signaling pathways and mouse behaviors. J. Biol. Chem. 299, 104583. 10.1016/j.jbc.2023.104583 36871761 PMC10139999

[B58] Zapata P. (1975). Effects of dopamine on carotid chemo- and baroreceptors *in vitro* . J. Physiol. 244, 235–251. 10.1113/jphysiol.1975.sp010794 235640 PMC1330755

[B59] Zhang M. Zhong H. Vollmer C. Nurse C. A. (2000). Co-release of ATP and ACh mediates hypoxic signalling at rat carotid body chemoreceptors. J. Physiol. 525, 143–158. 10.1111/j.1469-7793.2000.t01-1-00143.x 10811733 PMC2269919

